# Exploring the Secretomes of Microbes and Microbial Communities Using Filamentous Phage Display

**DOI:** 10.3389/fmicb.2016.00429

**Published:** 2016-04-07

**Authors:** Dragana Gagic, Milica Ciric, Wesley X. Wen, Filomena Ng, Jasna Rakonjac

**Affiliations:** ^1^Institute of Fundamental Sciences, Massey UniversityPalmerston North, New Zealand; ^2^Animal Science, Grasslands Research Centre, AgResearch Ltd, Palmerston NorthNew Zealand

**Keywords:** phage display, secretome, adhesins, metagenomics, next generation sequencing, bacteriophage

## Abstract

Microbial surface and secreted proteins (the secretome) contain a large number of proteins that interact with other microbes, host and/or environment. These proteins are exported by the coordinated activities of the protein secretion machinery present in the cell. A group of bacteriophage, called filamentous phage, have the ability to hijack bacterial protein secretion machinery in order to amplify and assemble *via* a secretion-like process. This ability has been harnessed in the use of filamentous phage of *Escherichia coli* in biotechnology applications, including screening large libraries of variants for binding to “bait” of interest, from tissues *in vivo* to pure proteins or even inorganic substrates. In this review we discuss the roles of secretome proteins in pathogenic and non-pathogenic bacteria and corresponding secretion pathways. We describe the basics of phage display technology and its variants applied to discovery of bacterial proteins that are implicated in colonization of host tissues and pathogenesis, as well as vaccine candidates through filamentous phage display library screening. Secretome selection aided by next-generation sequence analysis was successfully applied for selective display of the secretome at a microbial community scale, the latter revealing the richness of secretome functions of interest and surprising versatility in filamentous phage display of secretome proteins from large number of Gram-negative as well as Gram-positive bacteria and archaea.

## Introduction

Microbial secretome is a portion of the proteome comprising proteins that are targeted to the envelope of microbial cells, or are secreted into extracellular milieu. Microbial surface proteins mediate adhesion to other microbes or environmental surfaces, to facilitate colonization of an environment. They include secreted enzymes involved in breaking up various polymeric molecules to produce mono- or oligomeric foodstuffs that microbes can absorb and use as carbon and nitrogen sources for growth. Secretome harbors dominant targets of the host immune responses and are therefore of interest for vaccine development. In commensal and pathogenic bacteria these proteins are also involved in manipulating the innate and adaptive immune system’s signaling pathways. Although many surface proteins that mediate these functions have been identified in individual cultivated bacteria through genetic screens, identities of proteins that bind to specific targets of interest from archaea or from yet uncultivated microbes in complex microbial communities are largely unknown. Partial reason for this is that, with respect to primary sequence conservation, the secretome proteins are most variable group in microbial proteomes; it is rarely possible to predict the binding specificity of annotated secretome proteins using bioinformatics. In the absence of cultivation or genetic manipulation methods for a microbe, affinity screening of recombinant libraries is a suitable approach to identify proteins implicated in interactions of microbes with their hosts or environment. Phage display is a powerful combinatorial technology for affinity-selection of rare variants in vast libraries; due to the physical link between coding sequence inside the virion and protein displayed on the surface of the virion, large number of individual recombinant clones (up to 10^12^ per mL) can be affinity screened against complex baits of interest, such as tissues or extracellular matrix (ECM). Ff (f1, fd, or M13) phage whose virion proteins belong to the secretome and the virion itself is secreted out of *Escherichia coli*, is ideally suited for capture, correct folding and display of the secretome proteins. This article reviews the secretome and phage display technology applications in discovery of eubacterial and archaeal secretome proteins and emerging applications in functional metagenomics of the microbial communities’ secretomes.

## Eubacterial Secretome and Mechanisms of Secretion

The term ‘secretome’, coined by Tjalsma et al. (2000), was originally proposed to refer to both the secreted proteins and components of the protein secretion machineries in bacteria. Today, the secretome is broadly described as a subset of bacterial proteome, containing the extracellular proteome (exoproteome), released to the extracellular milieu and the surface-associated proteome, either exposed to the bacterial surface or intrinsic to the external side of plasma membrane and the cell wall, but excluding integral membrane proteins and proteins intrinsic to the internal side of the plasma membrane (Desvaux et al., 2009; Zhou et al., 2010).

Secretome proteins (e.g., receptors, transporters, adhesins, complex cell structures, secreted enzymes, toxins, and virulence factors) allow bacteria to interact with, and adapt to their environment. Bacterial secretory proteins are known to be involved in processes such as: provision of nutrients through recognition; binding, degradation, and uptake of complex extracellular molecules; communication between bacterial cells; detoxification of the environment; attachment to host cells and signal transduction; while in pathogenic bacteria they also play critical roles in virulence and immunogenicity (Walsh, 2000; Antelmann et al., 2001; Tjalsma et al., 2004; Wooldridge, 2009; Dalbey and Kuhn, 2012). Secretome proteins have been reported to occupy 10–30% of the total coding capacity of bacterial genomes (Wallin and Heijne, 1998; Kudva et al., 2013).

Secretome includes multi-protein surface appendages such as pili and flagellae, which have pivotal roles in bacterial attachment, horizontal gene transfer, and motility (Van Gerven et al., 2011). The pathways for membrane targeting are also “hijacked” by the filamentous phage or inoviruses, whose virion and assembly proteins belong to the secretome and which are secreted from bacteria without killing the host (Russel and Model, 2006; Rakonjac et al., 2011; Mai-Prochnow et al., 2015).

### Secretion Pathways of Bacteria

The cell envelope of Gram-positive bacteria consists of a single, cytoplasmic membrane and a cell wall, comprised of a thick peptidoglycan layer cross-linked with different molecules, such as capsular polysaccharides, cell wall teichoic acids, and proteins (Freudl, 2013). In contrast, Gram-negative bacteria are enveloped by inner (cytoplasmic) and outer membranes. The presence of two membranes defines an additional subcellular compartment (the periplasmic space), containing a thin meshwork of peptidoglycans. Some Gram-positive bacteria also have a distinctive thin granular layer (inner wall zone) between the membrane and the mature cell wall, equivalent to the periplasmic space in Gram-negative bacteria (Zuber et al., 2006).

In order to be anchored to the cell surface or released into the extracellular milieu, secretome proteins must be translocated across one or more biological membranes (Desvaux et al., 2006). Transport of proteins into or across biological membranes (translocation), catalyzed by membrane-bound proteinaceous transport machineries, is a universal event in the protein secretion mechanism, and it can occur several times during the course of secretion (Desvaux et al., 2009). Once a secreted protein is translocated across the outermost membrane, it can remain anchored (covalently or non-covalently associated with cell-wall components in Gram-positive bacteria or outer membrane components in Gram-negative bacteria), assemble into macromolecular structures on the cell surface (flagella, pili), be injected into host cells, or released to the extracellular milieu.

A remarkable array of systems for export of proteins have been described in Gram-positive and Gram-negative bacteria. Descriptive names are used in the nomenclature of systems involved in protein translocation across cytoplasmic membranes of both Gram-negative and Gram-positive bacteria, while an alphanumerical system has been adopted for naming protein secretion systems of Gram-negative bacteria (Desvaux et al., 2009).

Systems that are universally involved in protein translocation across the cytoplasmic membrane, and encoded in both Gram-positive and Gram-negative bacteria are: the conserved general secretion (Sec) system, YidC insertase, the twin-arginine translocation (Tat) system and hole-forming pathway *via* holins (Desvaux et al., 2009).

The Sec system is a major secretory pathway for protein insertion into the inner (cytoplasmic) membrane, and is conserved in all eubacteria. It is also ubiquitous in archaea, and the membranes of eukaryotic endoplasmic reticulum and chloroplasts (Szabo and Pohlschroder, 2012). This system also plays a key role in further transport of some proteins into the periplasmic space, outer membrane (e.g., lipoproteins and beta barrel proteins), or their assembly into the surface-associated structures (e.g., pili subunits). Furthermore, some of the components of the specialized secretion systems in Gram-negatives and their substrates (proteins transported *via* these secretion systems) are initially transported across the inner membrane by the SecYEG translocon (Beckwith, 2013; Kudva et al., 2013).

In bacteria, the Sec system is composed of the SecYEG translocon and three major accessory systems that target the secretome proteins to the translocon: SecB/A, SRP/FtsY, and YidC. SecYEG is an evolutionarily conserved heterotrimeric protein complex, and its SecY subunit forms an hourglass-shaped aqueous protein transport channel embedded in the inner membrane (Dalbey and Kuhn, 2012; Kudva et al., 2013). The translocon transiently interacts with different proteins during the transport process (e.g., SecA, FtsY, SecDF). SecA, a post-translational pathway motor protein accepts the substrate protein delivered by the cytosolic targeting factor SecB, and pushes it through the translocon in a stepwise and ATP-dependent manner (Lycklama et al., 2012). FtsY, the SRP-receptor, occupies the ribosome binding site (RBS) of SecY until its displacement by the translating ribosome during co-translational targeting (Kudva et al., 2013). The membrane-integrated SecDF chaperone uses proton-motive force to power ATP-independent protein translocation through the SecYEG channel (Tsukazaki et al., 2011).

In addition to universal secretion systems, Gram-positive bacteria possess Wss (WXG100 secretion systems), accessory Sec systems (SecA2-only and SecA2/SecY2 export pathways), flagella export apparatus (FEA), the fimbrilin-protein exporter (FPE), ABC protein exporter and Sec-dependent sortases. In Gram-positive bacteria, secreted proteins have several different fates. They are transported across the cytoplasmic membrane and then secreted into the extracellular milieu by SecYEG, Tat, holin, or Wss, in addition to being attached (covalently or non-covalently) to the cell wall using the sortase or assembled into the cell surface appendages *via* Sec pathway (e.g., cellulosomes or pili), *via* FPE (e.g., competence pseudo-pili), or *via* FEA (e.g., flagella).

Due to the added complexity of their cell envelope, at least two additional systems for targeting proteins to the outer membrane and eight additional systems for secretion of proteins outside of the cell have been described in Gram-negative bacteria. After Sec- or Tat- dependent translocation across the inner membrane, outer membrane-specific lipoproteins and unfolded β-barrel proteins are targeted to the outer membrane *via* the Lol pathway and β-barrel assembly machinery (BAM) pathway, respectively (Dalbey and Kuhn, 2012).

Secreted proteins targeted to the extracellular milieu, or to another cell, can be exported out of the cell directly, or by a two-step secretion process *via* type 1–6 secretion systems (T1SS–T6SS). In addition, the chaperone-usher system (CU or T7SS), the extracellular nucleation-precipitation mechanism (ENP or T8SS) system, as well as type IV pilus biogenesis (T4PBS) and tight-adherence (Tad) piliation systems are dedicated to exporting different types of pili subunits across the outer membrane (Chagnot et al., 2013).

The direct (Sec pathway-independent) secretion in Gram-negatives exports proteins through a contiguous secretion machinery spanning two membranes and the periplasm (T1SS, T3SS, T4SS, and T6SS systems). The two-step secretion process involves protein export to the periplasm by the Sec or, less frequently the Tat pathway, followed by export across outer membrane *via* T2SS, T5SS, T7SS, or T8SS systems. T1SS and T5SS are relatively simple systems involving few proteins, while T2SS, T3SS, T4SS, and T6SS are complex structures composed of large number of subunits, and spanning the entire bacterial cell envelope (Chagnot et al., 2013).

The filamentous phage assembly system follows a variant of the two-step secretion process, where all virion proteins are first inserted into the inner membrane, followed by the export-coupled assembly of the phage. The assembly is initiated by minor proteins pVII and pIX that interact with a specific phage genome sequence called the packaging signal, followed by rapid elongation by addition of major coat (pVIII) subunits (Rakonjac et al., 2011; Marvin et al., 2014). The virion proteins egress from the inner membrane to form a filament by attaching to double-helical DNA genome *via* few positively charged C-terminal residues that face the cytoplasm prior to assembly. The assembly process is catalyzed by phage-encoded inner membrane ATPase and requires ATP and proton motive force (Feng et al., 1997); the filament is released from the assembly site by two minor proteins, pIII and pVI (which themselves are integral membrane proteins) when the DNA is completely covered with pVIII (Rakonjac et al., 1999). The resulting filament has no phospholipids and has high temperature (70°C) and detergent (1% Sarkosyl) resistance (Crissman and Smith, 1984; Branston et al., 2013). Whereas over 70 filamentous phage of Gram-negative bacteria have been identified, only two were found in Gram-positives (Day, 2011).

### Secretion and Membrane Targeting Signals

The first stage in the secretome protein export is sorting and targeting of proteins to the cytoplasmic membrane, followed by membrane crossing and maturation/release of the translocated protein. The sorting process, through which proteins are directed to their specific subcellular compartments, is based on localization information contained in a short amino acid sequence that acts as a protein sorting signal (‘zip code’) governing protein traffic, transport, and localization in the cell (Blobel and Sabatini, 1971). Discrimination between secreted and cytoplasmic proteins is based on the presence of membrane-targeting sequences, such as signal sequences and transmembrane α-helices that are recognized by distinct secretory pathway-associated molecular chaperones, and are necessary for correctly targeting secreted proteins to the translocation pathway.

Most secreted proteins contain N-terminal signal sequences that are cleavable. The function and overall structure of the signal sequence, transmembrane alpha helix targeted to the SecYEG translocon, are conserved in all domains of life; however, these peptides lack primary sequence homology even within a species (Rusch and Kendall, 2007). Several types of signal sequences have been described: type I (classic) signal sequence, type II (lipoprotein) signal sequence, Tat signal sequence, type IV (pseudopilin-like) signal sequence and bacteriocin/pheromone signal sequence. Based on hydrophobicity and charge, most signal sequences have a conserved overall tripartite organization consisting of an hydrophobic core (h-domain), flanked by hydrophilic positively charged N-terminal region (n-domain) and a polar C-terminal region (c-domain) with cleavage/retention sites (Rusch and Kendall, 2007; Driessen and Nouwen, 2008; Zhou et al., 2008). However, the type IV and bacteriocin/pheromone signal sequences do not precisely follow such a structural layout (Zhou et al., 2008).

Signal sequences are usually removed during or shortly after their translocation across the membrane by several types of membrane-associated signal peptidases (SPases), which also have a role in quality control and regulated turnover of exported proteins (Dalbey et al., 2012). In bacteria, precursor proteins that are translocated through the Sec and Tat-pathways apart from pre-proteins (Lüke et al., 2009) are proteolytically processed by a ‘general’ type I signal peptidase (SPaseI) (Lüke et al., 2009; Auclair et al., 2012). Processing of the lipoprotein signal sequences is performed by type II lipoprotein signal peptidase (SPaseII). The lipoproteins are transported across the inner membrane in a Sec-dependent manner (Okuda and Tokuda, 2011). The Tat-dependent export of lipoproteins has only been demonstrated in streptomycetes (Thompson et al., 2010; Dalbey and Kuhn, 2012). The prepilin signal peptidase (SPaseIV) is responsible for processing proteins containing type IV signal sequence, such as pilins and related pseudopilins, that have mainly Sec-dependent export across the inner membrane (Peabody et al., 2003; Arts et al., 2007; Francetic et al., 2007).

**FIGURE 1:**
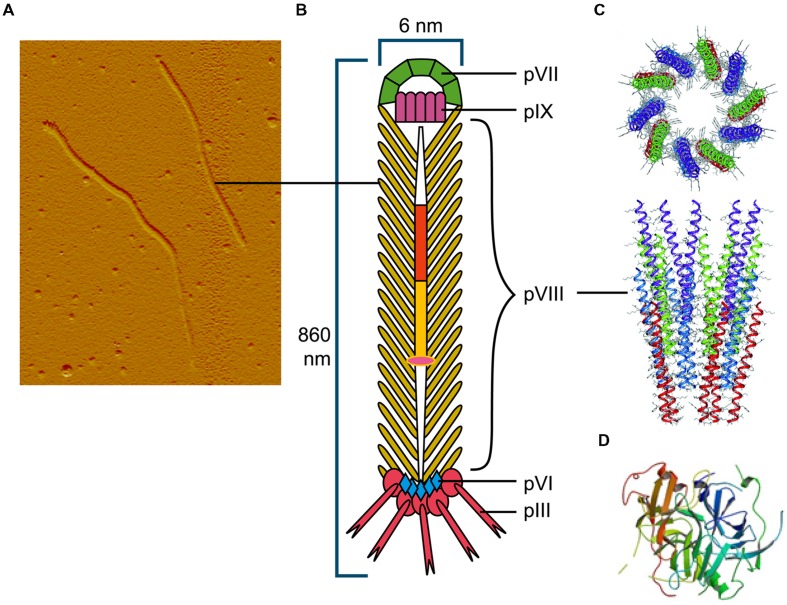
**The Ff bacteriophage structure and virion proteins used most commonly in phage display. (A)** Ff virion visualized by atomic force microscope (M. Russel and P. Model, sample prepared by J. Rakonjac). **(B)** Schematic diagram of Ff bacteriophage. **(C)** Ribbon representations (top and side view) of the pVIII coat protein (RCSB PDB database accession number 2cOw; (Marvin et al., 2006) arranged around bacteriophage single stranded DNA (not shown). **(D)** Ribbon representation of N1 and N2 domains of pIII (RCSB PDB database accession number1g3p; Lubkowski et al., 1998).

Among the Ff (f1, M13, or fd) filamentous phage virion proteins, major coat protein pVIII that forms body of the filament (**Figure 1**) has a type I signal sequence and is secreted by SecYEG translocons and, YidC (Samuelson et al., 2000); it is targeted to the inner membrane by a C-terminal transmembrane helix. The length of the mature pVIII protein (after signal sequence cleavage) is 50 amino acids. Once assembled into the viral particle, it is DNA-bound and helically arranged to form the shaft of the filament (**Figure 1**). The four minor proteins are all integral membrane proteins, however, only the largest, pIII (406 aa) has a type I signal sequence and its membrane targeting is SecYEG/SecAB-dependent (Chang et al., 1978). Minor protein pVI (112aa) has three predicted transmembrane helices, but no signal sequence. The remaining two minor proteins, pVII (33 aa) and pIX (32 aa) are very small and hydrophobic, each containing a transmembrane helix, but no signal sequences.

### Methods for Study of the Secretome

Mining bacterial secretomes is important for a range of applications, including identification of novel enzymes, understanding bacterial adhesion and their interactions with the environment, investigating pathogenic mechanisms, epitope mapping and identification of new vaccine candidates. Secretomes are traditionally studied *in vitro*, using biochemical and proteomics approaches, and *in silico*, using bioinformatic tools. Surface display screening methods and reporter fusion systems (Georgiou et al., 1997; Chen and Georgiou, 2002; Åvall-Jääskeläinen et al., 2003; Lee et al., 2003; Wernérus and Ståhl, 2004; Liu et al., 2013), as well as phage-display based systems (Rosander et al., 2002, 2011; Jacobsson et al., 2003; Bjerketorp et al., 2004), described in detail in section 3, have also been used for screening, identifying, and characterizing secretome proteins.

Secretomes are studied *in vitro* using high-resolution separation (2D gel electrophoresis and/or liquid chroma tography) of secreted or extracted membrane proteins, coupled with mass spectrometric methods for the identification of peptides and proteins in the sample (Yang et al., 2012). Biochemical approaches for elucidating the secretome of a microorganism allow direct functional characterization of identified proteins; however, they are very tedious and limited only to cultivable bacteria. Furthermore, construction of a proteome map of surface-associated and membrane proteins can be hindered by technical limitations of protein extraction from the membranes. In the absence of experimental data, a secretome can be deduced from a completely sequenced genome *in silico*, using bioinformatic tools for the prediction of secretome proteins based on their specific conserved features.

Computational methods for secretome protein prediction are based on weight matrices, sequence alignment or machine learning algorithms, and can be roughly grouped into global tools for subcellular protein localization prediction, and specialized tools for the prediction of signal sequences (Goudenège et al., 2010; Caccia et al., 2013). More sophisticated machine learning algorithms, based on neural networks and decision trees, support vector machines, Bayesian networks, HMMs, or their combination, are now more prominently used for discriminating secreted and non-secreted proteins (Zhou et al., 2008; Choo et al., 2009). During the training phase, typical signal and non-signal peptides are presented to the algorithm, and a classification model is subsequently built. Tools for signal sequence prediction such as SignalP (Petersen et al., 2011), LipoP (Juncker et al., 2003), TMHMM (Krogh et al., 2001), PRED-LIPO (Bagos et al., 2008), PRED-TAT (Bagos et al., 2010), SecretomeP (Bendtsen et al., 2005), and tools for subcellular protein localization prediction, such as PSORTb (Yu et al., 2010) or TargetP (Emanuelsson et al., 2000) belong to this class.

In bacteria, classically secreted proteins can be predicted based on recognition of the tripartite organization of their N-terminal, cleavable signal sequences, and conserved amino acid residues at the –3 and –1 positions relative to the cleavage sites. In addition to these, the lipobox of type II signal sequences and the Tat motif in Tat signal sequences are highly amenable to identification by bioinformatic tools, while transmembrane α-helices can be identified based on their hydrophobicity (Rahman et al., 2008; Goudenège et al., 2010). Recognition of the SPaseIV cleavage motif is not sufficient for the accurate detection of type IV signal sequences, since these have no tripartite structure like other Sec-dependent substrates. It was demonstrated that the specificity of searches for type IV pilin-like proteins may be enhanced by including additional search requirements, such as the presence of 14 sequential uncharged amino acid residues immediately after the cleavage motif or presence of a single transmembrane helix within 50 amino acid residues of the N-terminus, since true pilins contain only one transmembrane helix, typically close to the cleavage motif (Imam et al., 2011).

Cleavable N-terminal signal peptides of secreted proteins are readily distinguishable from longer hydrophobic N-terminal transmembrane helices of transmembrane proteins. In contrast, their discrimination from uncleaved N-terminal signal anchors, which tether some of these Sec-exported proteins to the membrane, is often problematic (Natale et al., 2008; Zhou et al., 2008; Caccia et al., 2013). However, tools such as SignalP 4.0 are trying to overcome this challenge by combining predictions of transmembrane protein topology with signal sequence identification.

The disadvantages of *in silico* secretome analysis is that it can be only applied to organisms with sequenced genomes; its accuracy depends on prediction algorithm performance, as well as on genomic annotation accuracy. Therefore, to improve the identification of secretome proteins, genomic predictions need to be integrated with transcriptomics and proteomics data (Caccia et al., 2013). The task of predicting the metasecretomes of complex environmental microbial communities is even more challenging. This is due to current limitations in the identification of complete genes *via* sequence-based metagenomics approaches from low-coverage metagenomic assemblies derived through next-generation sequencing of complex environmental microbial communities, often containing numerous closely related microbial species (Hess et al., 2011; Luo et al., 2011).

Despite the versatility of bioinformatics approaches in predicting the targeting sequences and cellular location of proteins, these methods are not capable of predicting exact functions that are essential for understanding vital and specific functions such as interacting with partners in the microbes’ surroundings. One experimental method that is well-suited for finding genes that fulfill the functions of interest is expression library screening and display of secretome proteins on the surface of bacteria or filamentous phage as recombinant fusion proteins. This method ensures that protein folding occurs under similar conditions to those where these proteins naturally fold – on cell surfaces. Bacterial cell surface display and yeast surface display are described elsewhere (Georgiou et al., 1997; Chen and Georgiou, 2002; Åvall-Jääskeläinen et al., 2003; Lee et al., 2003; Wernérus and Ståhl, 2004; Liu et al., 2013); this review focuses on filamentous phage display.

## Phage Display

**FIGURE 2:**
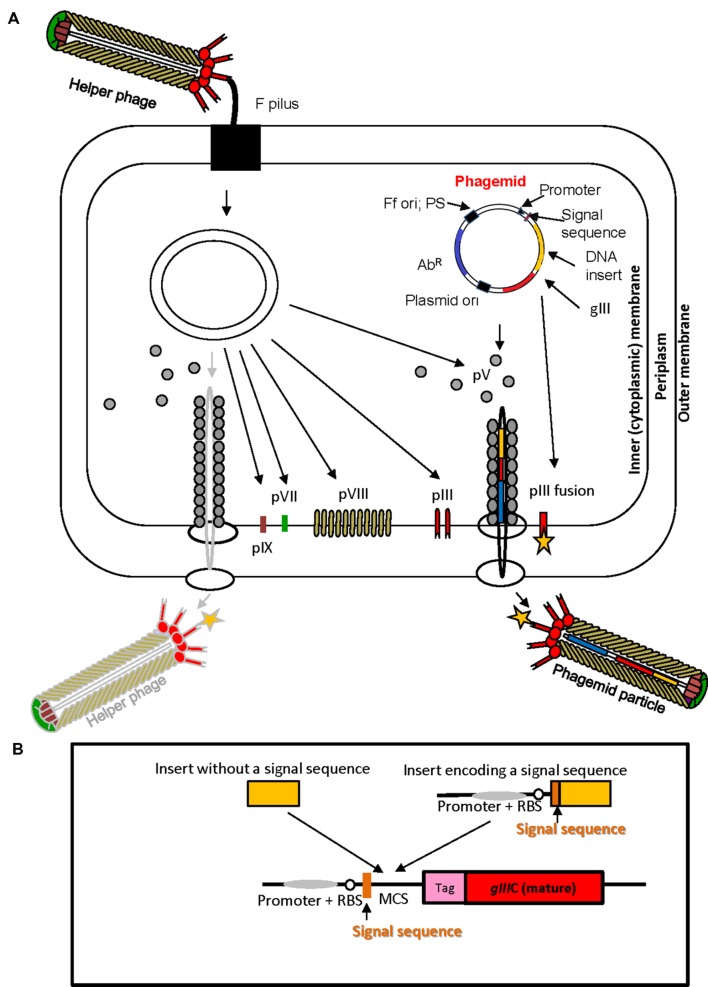
**Ff virion protein targeting, virion assembly, phagemid display system, and display cassette in phagemid vectors. (A)** In *Escherichia coli* phagemids can be replicated as plasmids or alternatively, in the presence of a helper phage, packaged as “transducing” or “phagemid” particles (PPs). Phagemid encodes phage protein pIII as a fusion partner for display. The resulting phagemid particles may incorporate either pIII derived from the helper phage (red lollipop-like structure) or the polypeptide-pIII fusion protein (red lollipop-like structure decorated with a yellow star), encoded by the phagemid. Ab^R^, antibiotic resistance marker, Ff ori; PS, filamentous phage origin of replication and packaging signal; Plasmid ori, plasmid origin of replication; gIII, gene III; pIII, pV, pVI, pVII, pVIII, and pIX, filamentous phage proteins. Phagemid particles are produced at a 10–100-fold excess over the helper phage (denoted by solid vs. faded lines for phagemid particle vs. helper phage. **(B)** Typical phage display cloning cassette in a phagemid vector: promoter, a ribosome binding site (RBS), signal sequence (commonly used PelB signal sequence from *Erwinia carotovora* (Lei et al., 1987); multiple cloning site (MCS), affinity tag (tag), and sequence encoding the mature portion (C-terminal domain) of pIII (*gIII*C; required for assembly of the fusion into the virion). Two types of inserts derived from fragmented bacterial or archaeal genomic DNA are shown above the cloning site. Insert without signal sequence, in order to result in displayed peptide, has to correspond to a CDS that is in frame with the upstream signal sequence and downstream *gIII* (encoding the C-terminal domain). It is typically truncated at the 5′ and 3′ ends to avoid stop codons that terminate translation. A second type of insert, that contains signal sequence, can be displayed if the CDS is truncated at the 3′ end, and is in frame with *gIII*. The latter type of inserts typically carries its own promoter and RBS. *E. coli* host strains that contain suppressor mutations (such as *supE44*) can read through *amber* stop codons at 50% efficiency and result in display of fusions that include this codon.

The physical link between phenotype and genotype of a (poly) peptide displayed on the surface of the virion, the high replication capacity of bacteriophage and subsequent affinity selection are the elements that underpin phage display technology. In phage display, a very large repertoire of recombinant phage particles displaying (poly) peptides can be generated (10^12^–10^13^ different clones) at very low cost using simple methods of microbiology and molecular biology. Nucleotide sequence repertoires such as cDNAs, synthetic oligonucleotides, genomic DNA fragments derived from single organisms or metagenomes, and mRNAs are cloned directly into phage display vectors. Display of peptides encoded by cloned sequences is achieved by translational fusion of a protein or a library of proteins of interest to any of the five structural virion proteins, pVIII, pIII, pVI, pVII, or pIX at N- or C-terminus, although the pIII and pVIII proteins are used most frequently (Russel et al., 2004; Kehoe and Kay, 2005). Peptides fused to the Ff virion proteins fold in the periplasm of *E. coli* (**Figure 2A**), therefore display on filamentous phage is suitable for surface and secreted proteins.

In contrast to Ff phage proteins, “tailed” bacteriophages λ, T7, T4, and P4 fold in *E. coli* cytoplasm and are therefore most commonly used for the display of cytoplasmic proteins. The distinction between cytoplasmic and extracytoplasmic environments mostly refers to oxidative state of Cys residues; which are nearly always reduced in cytoplasmic (or nuclear) proteins, whereas they are oxidized in the periplasm of Gram-negative bacteria or membranous organelles in eukaryotic cells. The readers are referred to several reviews for the latest developments in tailed phage display (Krumpe et al., 2006; Beghetto and Gargano, 2011; Gamkrelidze and Dabrowska, 2014).

Despite the enormous body of reports describing display of a variety of proteins on pIII and pVIII display platforms, there are still many proteins that are recalcitrant to functional display due to host restrictions such as codon usage, protein folding and toxicity to the *E. coli* host. To some degree folding constraints can be alleviated by introducing changes to the host periplasm environment, for example using *ΔdsbA E. coli* strains that do not form S–S bridges between Cys residues to allow folding and display of cytoplasmic proteins, or co-expressing with periplasmic chaperones such as Skp and FkpAto facilitate folding of the fusion counterpart (Hayhurst and Harris, 1999; Gunnarsen et al., 2013), or using alternative signal sequences or translocation routes *via* SRP-dependent or indirect Tat phage display systems (Steiner et al., 2008; Speck et al., 2011).

The non-specific binding to matrices where bait is immobilized can be decreased by “wrapping” the phage in desired charge that is repulsive to the matrix. For example, fusing Lys_8_ peptide to all major coat protein subunits can be used for positively charged surfaces (Lamboy et al., 2008). To overcome limitations of using only 20 amino acids for the construction of peptide or protein libraries, phage display technology was adapted to incorporate unnatural amino acids by using host strains that express mutant tRNAs and aminoacyl-tRNA synthetases (Tian et al., 2004; Bratkovic, 2010; Bernard and Francis, 2014).

Recently, the scope of phage display extended toward the display of polypeptides containing posttranslational modifi cations, including phosphorylation, phosphopantetheinylation (Yen and Yin, 2007) and glycosylation (Celik et al., 2010; Dürr et al., 2010; Ng S. et al., 2015). As glycosylation is the most frequent posttranslational modification [>50% of total eukaryotic proteins and an increasing number of archaeal and bacterial proteins (Abu-Qarn et al., 2008)], modification of displayed proteins has promising future applications.

### Filamentous Phage Display Types

There are two general types of filamentous phage display based on whether the library is constructed in Ff phage vectors or in specially modified plasmids called phagemids in conjunction with helper phage. The advantage of a phage vector system is in its simplicity. The library constructed in a simple Ff phage vector results in fusion gene products being displayed by all copies of a particular virion protein. Given that some protein fusions in a library are likely to interfere with assembly and infectivity, they are reported to be counter-selected during the phage life cycle and are lost from the library (Rodi and Makowski, 1999; Derda et al., 2011). Insertion of a second copy of a particular virion protein-encoding gene is one strategy to overcome the interference (Barbas et al., 2001). For pIII fusions, the inter-domain display is solution to prevent censorship due to proteolytic degradation of peptides (Tjhung et al., 2015).

Another type of phage display system that allows more flexibility and provides independence from phage assembly in the amplification phase is based on phagemid vectors (**Figure 2**). Phagemids are plasmid vectors that contain the Ff origin of replication, packaging signal, a plasmid origin of replication, and a selective (antibiotic resistance) marker. Phage display phagemid vectors in addition contain a display cassette, allowing construction of fusion to a virion protein. The Ff origin of replication, packaging signal and the display cassette allow packaging of the phagemid genome into phage-like “phagemid particles” (PPs) and display of phagemid-encoded protein fusion on the surface of the particle (**Figure 2**). A helper phage is the obligatory source of proteins necessary for replication from Ff origin and assembly of a complete virion.

Helper phage typically have an interference-resistant Ff origin of replication and/or truncated packaging signal, or additional (plasmid) origin of replication, allowing preferential replication and packaging of the phagemid single stranded DNA so that the majority (~90%) of secreted virions are PPs rather than the helper phage (Barbas et al., 2001). Helper phage that encode all virion proteins and contribute them to the PPs are herein referred to as a wild-type helpers. The phagemid/wild-type helper system typically results in monovalent display of recombinant protein fusions to minor virion proteins (pIII, pVII, pIX, and pVI); i.e., PPs are mosaic for recombinant and wild-type capsid proteins produced by phagemid encoding the fusion protein and by a helper, respectively (**Figure 2A**). To increase the copy number of displayed fusion proteins, helper phage containing deletion of the gene encoding the virion protein that is expressed from the phagemid can be used. In the case of most frequently used phagemid vectors that encode virion protein pIII, helper phage that carry deletion of corresponding gene (e.g., *ΔgIII*) are used. The obtained virions exhibit polyvalent display (Griffiths et al., 1993; Rakonjac et al., 1997; Joo et al., 2008). Additional refinements of some phage display phagemid vectors are peptide tags (e.g., c-myc or E-tag), followed by an *amber* stop codon between the insert and downstream pIII. These additions allow a soluble version of foreign protein to be produced if phagemids are transformed into an appropriate *E. coli* strain that does not contain an amber codon suppressor (Hoogenboom et al., 1991).

### Affinity Selection of Binding Targets – Bio-Panning

**FIGURE 3:**
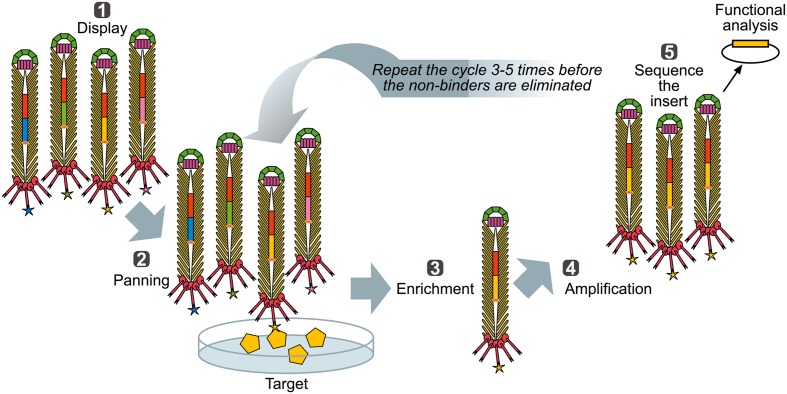
**Biopanning – a basic selection for binding peptides.** (1) Display: Filamentous phage displaying variants of proteins/peptides/antibodies is created, cloned into phage, or phagemid vectors as fusions to a coat protein gene(s) and displayed on the surface of the virions. (2) Panning: The phage library displaying variant peptides or proteins (different-color stars) is exposed to immobilized ligand (yellow pentagon) and phage with appropriate binding specificity is captured (yellow star). (3) Enrichment: Non-binding phages are washed off and bound phage(s) is (are) eluted by conditions that disrupt the peptide-ligand interactions, leading to enrichment for a specific binder. (4) Amplification: Eluted phage is then amplified by infection of a suitable *E. coli* strain. This amplified phage population is greatly enriched in recombinant phage clones displaying peptides that bind to the target. The biopanning steps (two to four) are repeated for several (three to five) rounds, ultimately resulting in a clonal population of recombinant phages that bind to the target used for affinity panning of the library. Captured putative binder can then be identified by sequencing (5) and functionally analyzed.

When a library of fusion proteins is constructed and displayed on the phage surface, a recombinant phage clone displaying a certain binding affinity can be selected from the majority of other (non-binding) recombinant phages present in a library, by an affinity selection procedure known as bio-panning (**Figure 3**) (Parmley and Smith, 1988). Through successive rounds of binding, washing, elution, and amplification, an originally very diverse phage display library, up to 10^12^ variants (Schier et al., 1996) is increasingly enriched for the phage library clones with a propensity to bind to the target molecule. Ultimately, monoclonal phage populations with desired specificities can be identified using sequencing and analyzed using affinity binding assays.

Biopanning has potential to identify proteins that bind to enormous diversity of ligands, from extremely complex, including the whole animal (Arap et al., 1998), *ex vivo* tissues (Antonara et al., 2007), complex mixture of organisms (Ng F. et al., 2015) and whole cells (Fevre et al., 2014) to very simple, such as purified proteins, peptides, nucleic acids, carbohydrates (Zwick et al., 1998; Rodi and Makowski, 1999) or inorganic powders (Mao et al., 2004; Ploss et al., 2014). The outcome of biopanning, however, depends on library complexity (or primary size, equivalent to the number of different variants or recombinant inserts), functionality of displayed fusions and affinity of interaction with the bait used in the biopanning. For isolation of high-affinity binders, monovalent phage display libraries are usually used because they allow selection based on strong affinity. In contrast, multivalent display, in particular on the major coat protein (pVIII), is more suitable for selection of low-affinity binders since the avidity of the virion compensates for low affinity of individual peptides (Shallom and Shoham, 2003).

### Phage Display Applications in Functional Identification of Microbial Secretome Proteins

Microbial interactions with the environment have beneficial or detrimental effects on microorganisms involved in interactions or/and the host. In general, these interactions are mediated *via* microbial secretome proteins that interact with receptor molecules on host cells or other microbes, or with the host cell surface or proteins of the host signaling pathways involved in the immune response. Phage display has been used extensively for identification of bacterial proteins that interact with the host surfaces or are dominant immunogens recognized by host antibodies (Mullen et al., 2006). Bacterial and more recently archaeal phage display libraries are constructed in phagemid vectors, as fusions to pIII or pVIII. Given the lack of introns in bacterial coding sequences, fragmented genomic DNA is used for construction of libraries. These are ligated to a vector that contains a multiple cloning site (MCS) between the signal sequence and the mature portion of gene III or VIII (**Figure 2B**). If a fragment is ligated such that the coding sequence is in the same direction and frame with the vector sequence encoding both the upstream signal sequence and downstream mature portion of protein, the encoded peptide will be displayed on the surface of phage. This type of fusion can display both the cytosolic and secretome proteins. Alternatively, if a CDS segment encoding a signal sequence is inserted in frame with the mature portion of pIII or pVIII (but not the vector-encoded signal sequence), it is also displayed on Ff. This type of recombinant clone will often contain the native promoter and RBS derived from the genomic DNA. Given the higher probability of a single in-frame joint than that of double, the secretome clones are expected to be reasonably frequent in the shot-gun filamentous phage display libraries. All clones that have inserts whose translated peptide is displayed on the phage surface can be pre-selected by virtue of peptide tags engineered into the mature portion of pIII (**Figure 2B**). Both types of fusions can be selected in the shot-gun phage display library screenings (Gagic et al., 2013; Ng F. et al., 2015).

Phagemids that rely on pIII for bacterial phage display library construction contain only the coding sequence for the C-terminal domain. The reason for this is that the pIII N-terminal domain expression results in resistance of phagemid-containing cells to helper phage infection, if a promoter sequence is present in the insert (Jankovic et al., 2007).

In the past two decades, particularly in the area of infectious diseases, phage display has proven to be a powerful technology for identification of genes encoding bacterial adhesins and characterization of adhesin domains that mediate interactions with host cells and ECM components such as fibronectin, fibrinogen, collagens, vitronectin, laminin, and heparin sulfate (Mullen et al., 2006). In pathogenic bacteria, microbial adhesion precedes colonization and internalization into the host cell and increases resistance to host defenses. Global spread of antibiotic-resistant pathogenic bacteria commands increased efforts to find new antigenic epitopes for vaccine development and alternative therapies that include interference with microbial adhesion or prevention of immune manipulation by pathogenic bacteria. Shot-gun filamentous phage display (display of random fragments of bacterial genomic DNA) was first exploited by Jacobsson and Frykberg (1996) to identify *Staphylococcus aureus* proteins that interact with ECM components and host serum or plasma. Following this initial publication many genes encoding proteins involved in host-microbial interactions from a number of bacterial species have been identified using phage display [**Table 1**; (Ausmees et al., 2001; Lauterbach et al., 2003; Benedek et al., 2005; Mullen et al., 2006; Antonara et al., 2007; Posadas et al., 2012; Evangelista K. V. et al., 2014]. A shot-gun phage display library of *Borrelia burgdorferi* was used for the *in vivo* screening for potential adhesins, resulting in discovery of at least five new adhesion proteins (Antonara et al., 2007). Phage display allows not only identification of a gene of interest, but also domains that mediate binding, by identifying a consensus among the library inserts that bind to a bait. For example, this approach was used to identify and map von Willebrand factor binding protein from pathogenic *Staphylococcus aureus* (Bjerketorp et al., 2002) and novel neutrophil-binding proteins of *S. aureus* that inhibit neutrophil recruitment to the site of infection (Fevre et al., 2014). Phage display has recently been used for identification of antigenic determinants of *Salmonella enterica sv.* Typhimurium (Meyer et al., 2012).

**Table 1:** Bacterial adhesins identified using phage display technology.

Organism	Ligand	Gene product or ORF encoding for bacterial adhesin	Reference
**Pathogenic bacteria**
*Helicobacter pylori*	Plasminogen	PgbB	Jönsson et al., 2004
*Staphylococcus aureus*	Human IgG	Sbi	Jacobsson and Frykberg, 1995
	von Willebrand factor	vWBp	Bjerketorp et al., 2002
	Platelets	FnBPA, FnBPB	Heilmann et al., 2002
	Fibronectin	FnBPA	Ingham et al., 2004
	Neutrophils	Selx	Fevre et al., 2014
	Neutrophils	SSL6	Fevre et al., 2014
	Fibronectin	Embp	Williams et al., 2002a
*Staphylococcus epidermidis*	MC3T3-E1 osteoblast cell line and Fibronectin	FnBPA, FnBPB	Williams et al., 2002b
	Fibrinogen	Fbe	Nilsson et al., 1998
*Staphylococcus lungdunensis*	Fibrinogen	Fb1	Nilsson et al., 2004a
	von Willebrand factor	vWbl	Nilsson et al., 2004b
*Streptococcus agalactiae*	Fibronectin	ScpB	Beckmann et al., 2002
	Fibrinogen	FgagV1, FgagV2, FgagV3	Jacobsson et al., 2003
*Streptococcus dysagalactiae*	Fibrinogen	DemA	Vasi et al., 2000
*Streptococcus equi*	Fibronectin	FnBP	Lindmark and Guss, 1999
*Haemophilus influenzae*	Bovine serum albumin	Hi0367	Mullen et al., 2007
*Actinobacillus pleuropneumoniae*	Bovine serum albumin	ORF16	Mullen et al., 2007
*Pasteurella multicida*	Fibronectin	Pm1665	Mullen et al., 2007
*Aggregatibacter actinomycetemcomitans*	Bovine serum albumin	ORF10	Mullen et al., 2007
*Salmonella enterica sv.* Typhimurium	Antisera from convalescent pigs	16 antigenic peptides	Meyer et al., 2012
*Leptospira interrogans*	Heparin	LigB	Ching et al., 2012
	EA.hy926 endothelial cells	LIC10508	Evangelista K. et al., 2014
	EA.hy926 endothelial cells	LIC13411	Evangelista K. et al., 2014
	EA.hy926 endothelial cells and VE-cadherin	LIC12341	Evangelista K. et al., 2014
	EA.hy926 endothelial cells and VE-cadherin	LIC13411	Evangelista K. et al., 2014
*Brucella suis*	Fibronectin	BmaC	Posadas et al., 2012
	Fibronectin	BRA0095	Posadas et al., 2012
	Fibronectin	BRA0175	Posadas et al., 2012
*Mycobacterium tuberculosis*	TB patient sera	LpqB	Liu et al., 2011
	TB patient sera	CpsA	Liu et al., 2011
	TB patient sera	PapA2	Liu et al., 2011
*Borrelia burgdorferi*	Mammalian cells	P66, OspC, VlsE, Lmp1, BmpD, OspF homolog, ErpK, ErpL, OspG	Coburn et al., 2013 and references therein
*Campylobacter jejuni*	Holo- and apo-lactoferin	LimC	Abruquah, 2009
*Yersinia pestis*	Laminin	Pla	Benedek et al., 2005
**Non-pathogenic bacteria**			
*Lactobacilllus rhamnosus* HN001	HN001 cells	SpcA	Gagic et al., 2013
	SpcA	SpcB	Gagic et al., 2013
*Lactobacillus casei* BL23	Collagen	XpkR, LCABL_01820	Munoz-Provencio and Monedero, 2011
	Fibronectin	Ps356 endolysin-homolog	Munoz-Provencio and Monedero, 2011
*Rhizobium leguminosarum* bv. *trifolii*	*Rhizobium leguminosarum* bv. *trifolii R200 cells*	RapA1	Ausmees et al., 2001
*Bifidobacterium longum* VMKB44	HT-29 epithelium cells	ABC transporter – BL0155	Shkoporov et al., 2008

Most bacteria in the environment, as well as commensal bacteria that colonize eukaryotic organisms, use adhesins for interactions with inorganic environment, other microbes and multicellular hosts. In comparison to the plethora of adhesins from pathogenic bacteria that we recognize today, adhesins from non-pathogenic bacteria are as numerous; however, their interacting partners are largely unknown. In one example of phage display application in symbiotic bacteria, it was used to identify and map the cell-surface-associated agglutinin (RapA) from legume root nitrogen-fixing bacterium, *Rhizobium leguminosarum* (Ausmees et al., 2001). Another large bacterial group of interest are probiotics. Although benefits of probiotic bacteria to human health are still debated, adhesion to gut mucosal surface is a mechanism by which they can persist in gut and also may preclude attachment of enteropathogenic bacteria and viruses to the host’s intestinal epithelial cells, possibly leading to beneficial effect. Several proteins from probiotic *Lactobacillus casei* binding to collagen and fibronectin have been identified (Munoz-Provencio and Monedero, 2011), as well as a *Lactobacillus rhamnosus* surface protein complex have been identified by affinity-screeing of phage display libraries (Gagic et al., 2013). Surface proteins from probiotic bacteria *Lactobacillus reuteri* and *Lactobacillus rhamnosus* at genome level have also been displayed using secretome-specific phage display method to obtain clone banks of displayed surface and secreted proteins suitable for binding and functional assays (Wall et al., 2003; Jankovic et al., 2007; Gagic et al., 2013).

Microbe-microbe interactions in complex environments and secretome proteins involved in these interactions are another research area of interest in recent years. For example in fermentative forestomach (rumen) of ruminants protozoa and specific group of archaea, methanogens, can be found in association. This interaction may facilitate hydrogen transfer from protozoa (hydrogen producers) to archaea, which use hydrogen to produce methane (Ushida et al., 1997; Belanche et al., 2014). Recently Ng F. et al. (2015) used shot-gun phage display in a phagemid/helper phage platform to identify a secretome protein Mru_1499, from rumen methanogenic archaeon *Methanobrevibacte rruminantium* M1 (Leahy et al., 2010) as a binder to a broad range of rumen protozoa and rumen bacterium *Butyrivibrio proteoclasticus*, suggesting a broad adhesion spectrum for this protein. Interestingly, the library insert contained an archaeal signal sequence and the 5′ portion of the Mru_1499 gene fused to the mature portion of pIII. Display and selection of this recombinant phagemid from the library therefore showed that archaeal signal sequences are functional in *E. coli*. Protozoa isolated from the sheep rumen were used as bait for the library panning to identify Mru_1499, and in turn the phagemid particles displaying Mru_1499 were used as bait to identify, among rumen protozoans, those that interact with this protein, by sequencing the 18S rRNAgene from the captured protozoa (Ng F. et al., 2015).

### Selective Display of Bacterial Secretome

**FIGURE 4:**
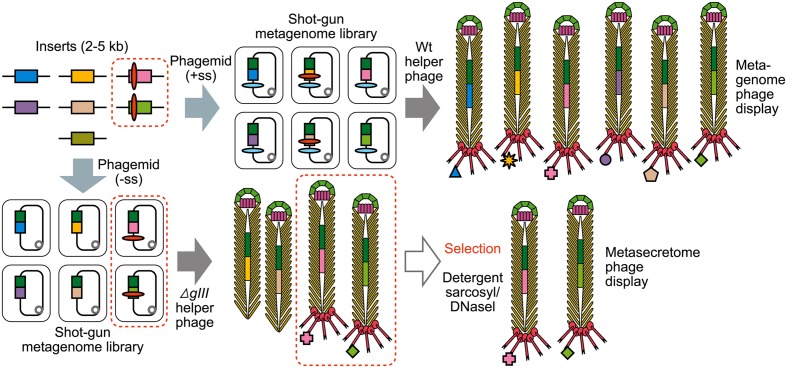
**The construction of shotgun metagenomic and metasecretome filamentous phage display libraries.** Metagenomic DNA is randomly sheared and cloned into phagemid that contains signal sequence (+ss) or as in case for metasecretome into phagemid without signal sequence (-ss). In both cases constructed metagenomic inserts contain endogenous signal sequences, represented by red ovals. Depending on helper phage used [wild-type (wt) or *gIII-*deleted helper phage] for aid in replication and assembly of recombinant virions, the library will contain virions displaying the whole metaproteome (metagenome phage display) or it will consist of virions capped by insert-pIII fusion proteins (signal sequence-positive clones) that are resistant to sarkosyl (Sarkosyl^R^, virions inside the dotted line) and uncapped virions (signal sequence-negative clones) that are sensitive to sarkosyl (Sarkosyl^S^). Sarkosyl resistance is used as a basis for selection in metasecretome phage display. Single stranded DNA (ssDNA) purified from Sarkosyl^R^ virions after the selection can be used to obtain the amplified metasecretome plasmid library for preliminary assessment of metasecretome diversity by next-generation sequencing [Taken from (Ciric et al., 2014) with permission].

A standard strategy for identifying targets for vaccine development in the genomics era is bioinformatic identification followed by expression of each individual bacterial secretome protein. This involves cloning and expression of up to several hundred surface proteins and testing their immunogenicity. Selective display of the secretome was proposed as an alternative to this path to allow, in one cloning step, the production of a library and/or clone bank of those recombinant phagemids that display surface-associated and secreted proteins. Secretome-selective phage display methods exploit the requirement of a signal sequence to target a fusion protein that is in frame with the mature portion of pIII to *E. coli* inner membrane (**Figure 4**). The phagemids used in these conditional display strategies contain C-terminal domain of pIII without a signal sequence. For assembly of signal sequence-encoding inserts (Rosander et al., 2002) used *gIII*-positive helper phage and for selection, biopanning based on the presence of a vector-encoded affinity tag incorporated into the fusions that were displayed on the phagemid particles thanks to a functional signal sequence. In the second method, a helper phage with *gIII* deletion was used for assembly of the particles (Jankovic et al., 2007). Here, the selection was based on removal of pIII-deficient phagemid particles which are structurally unstable and easily disassembled by detergent sarkosyl (Rakonjac and Model, 1998; Rakonjac et al., 1999). This approach was further used to identify six secretome proteins from *Mycobacterium tuberculosis* by subtractive panning between the sera of *M. tuberculosis* patients and BCG-vaccinated healthy subjects (Liu et al., 2011), three of which have not been identified prior to this study and which therefore were novel vaccine candidates. Interestingly, the breakdown of targeting sequences that were able to guide the fusion to the inner *E. coli* membrane and allow assembly of the virion included not only type I signal sequences, but also type II (lipoprotein) and type IV pre-pilin signal sequences as well as transmembrane helices. Moreover, a few “moonlighting” proteins that do not contain SecYEG-dependent signal sequences were identified among the selected fusions (Jankovic et al., 2007; Liu et al., 2011). Together with chance identification of an archaeal-signal-sequence-driven secretome protein fusion reported by Ng F. et al. (2015), the findings that signal sequences and transmembrane helices from Gram-positive bacteria, Mycobacteria and Archaea guide the pIII fusion protein to correctly insert into the inner membrane of *E. coli* and be processed to be displayed on the surface of the virion, indicate great plasticity in processing of the SecYEG-translocated pIII fusions. With respect to transmembrane helices and signal sequences other than type I, this process most likely involves periplasmic proteases other than signal peptidases, and in the case of moonlighting proteins it could involve SecYEG- and signal-sequence-independent translocons such as ABC transporters. These hypotheses remain to be experimentally verified.

Although in some cases secretome display was used for screening of libraries to identify binders to targets of interest (Fevre et al., 2014), this method is very strongly biased toward display of N-terminal portions of the secretome proteins, thereby eliminating binding domains encoded toward the C-terminal end of the large adhesins. Furthermore, with a few exceptions listed above this strategy is not expected to display important SecYEG-independent virulence factors, such as those secreted by the Type 1, 3, 4, and 6 secretion systems, which are predominant in enteric pathogens including *Yersinia*, *E. coli*, and *S. enterica* (Chagnot et al., 2013). Therefore, shot-gun phage display is more suitable than secretome display for screening to identify binding domains for novel ligands.

### Filamentous Phage Display in Functional Metagenomics

It is clear from the vast number of research reports that phage display technology has been used successfully to identify and characterize microbial proteins that interact with complex and simple targets as well as to build clone banks of secretome proteins from single organisms. Currently, the large volumes of data generated from fast improving next-generation-sequencing (NGS) technologies and metagenomics research are mainly used for cataloging and comparing the composition of microbial communities. An interesting question is whether the power of phage display and NGS could be combined to identify specific ligand-binding or representative (meta) secretome protein libraries from complex microbial communities.

Metagenomic approaches for analysis of microbial DNA recovered directly from the environment (Handelsman, 2004) have been used over the past decade to uncover extraordinary functional potential of complex microbial communities. Major strategies applied for identification of genes encoding “high-value targets” (enzymes for synthesis of novel bioactive molecules or novel biological activities) from metagenome are sequence-based bioinformatics analyses of the gene content, or function-based, by functional screening of metagenomic expression libraries (Tuffin et al., 2009; Ekkers et al., 2012; Culligan et al., 2013). Sequence-based metagenome mining aims to identify *in silico* high-value target gene or gene cluster candidates for heterologous expression, while function-based screens aim to phenotypically detect biosynthetically or enzymatically active clones (Charlop-Powers et al., 2014). Use of next generation sequencing (NGS) technologies in metagenomics enables significantly higher resolution and throughput in gene discovery from environmental microbes compared to traditional genomic approaches.

Genetic diversity of most microbial communities is tremendous. This implies an immense DNA sequencing volume is required to find, very sparse high-value target genes and gene clusters. A variety of enrichment strategies ranging from the whole-cell approach based on nutritional, chemical or physical selection, to increasing the frequency and diversity of genes likely to encode novel bioactive molecules, have been applied to increase the screening hit rate and speed up the process of gene discovery using functional metagenomic approaches (Cowan et al., 2005; Banik and Brady, 2010; Xing et al., 2012). DNA stable-isotope probing (Chen and Murrell, 2010; Mazard and Schäfer, 2014), complementation (Charlop-Powers et al., 2012), affinity capture and a number of strategies based on PCR and/or DNA hybridization, such as suppression subtractive hybridization (Galbraith et al., 2004; Meyer et al., 2007; Meiring et al., 2010), differential display, sequence tag interrogation approach (Owen et al., 2013) and metagenome arrays (He et al., 2007; Park et al., 2008; He et al., 2010) have been explored for enriching environmental DNA samples for genes of interest.

Although successful affinity screening of metagenomic Ff phage display libraries is yet to be reported, the precedents for this have been published in the form of selective metasecretome display and NGS-facilitated phage display library screening.

### Phage Display Combined with Next-Generation Sequencing

The traditional approach for screening of phage display libraries, consisting of 3–5 rounds of affinity selection (biopanning) followed by sequencing of inserts from limited number of clones (**Figure 3**), is laborious and unsuitable for screening of metagenomes of diverse microbial community or complex antibody repertoires of higher organisms. In contrast, use of NGS technologies, typically delivering >10^6^ of sequencing reads, is well suited for high-throughput exploration of the diversity of phage binding variants enriched after one or two rounds of panning on ligand of interest. The NGS analysis after limited panning allows high sequence coverage of binding variants, thereby overcoming a problem of competition between high-affinity and low-affinity binders and reducing false positive hits that often arise as a result of binding to non-bait materials present in the selection system (e.g., plastics, BSA) and propagation advantages (Vodnik et al., 2011).

Dias-Neto et al. (2009) proposed the use of NGS in combination with real-time PCR to improve phage quantification and analysis of the library inserts encoding phage-displayed variants. The authors adapted pyrosequencing for deep-sequencing of amplicons derived from phage ssDNA, retrieved directly after two rounds of panning of combinatorial library of random heptapeptides *in vivo* from four human tissues biopsies, using primers flanking the library insert within the fusion. This approach was applied to obtain sequencing reads directly from tissue-selected and unselected phage display libraries (library before and after panning) in a single run.

Di Niro et al. (2010) screened an ORF-filtered (Zacchi et al., 2003) cDNA phage display library, obtained from mRNA derived from several human cell lines, to identify proteins interacting with tissue transglutaminase 2, an enzyme implicated in different pathological conditions. In this study the authors combined phage display and NGS, enabling at least two orders of magnitude increase in the number of affinity-selected clones compared to traditional affinity screening, with two rounds of panning, to achieve an optimal balance between high numbers of positive clones and broad diversity. This approach led to identification of a “landscape” of binding variants from the phage display library, three of which had been previously characterized as transglutaminase 2-interacting proteins, with the remainder being novel proteins that were subsequently confirmed by functional assays. Based on the ranking of the most frequently selected ORFs within the selected phage population, detected through the high-throughput sequencing, it was estimated that at least 1000 clones would have to be picked and analyzed using traditional screening approach in order to capture the top five most frequent clones after two rounds of selection with 99% probability.

Recently, t Hoen et al. (2012) demonstrated that NGS can improve and accelerate finding of specific binders by screening of the phage display libraries, while reducing the number of false positive hits. Illumina platform was used to compare diversity of Ph.D.-7 M13 peptide phage display library before panning and phage display library retrieved after each of several rounds of panning on osteoblasts, performed to select heptapeptides that mediate binding to and uptake into osteoblasts. It was demonstrated that deep sequencing of the phage pool obtained after the first round of biopanning on osteoblasts was sufficient to identify positive hits. Selection of peptides with high binding to and uptake into osteoblasts was confirmed by confocal microscopy and live cell imaging. In addition, by sequencing the starting phage display library before screening (after one round of amplification in bacterial host), authors identified propagation advantage as an important source of false positive hits (t Hoen et al., 2012).

Due to the high diversity of antibody phage display libraries (typically between 10^7^ and 10^11^ variants) and observed correlation between the size of the repertoire and the antibody affinities isolated from it (Hust and Dubel, 2004), filamentous phage display coupled with NGS is routinely used for in-depth characterization of immunoglobulin antibody repertoires of different organisms and high-throughput screening of ligand-antibody interactions (Ravn et al., 2010; Reddy et al., 2010; Saggy et al., 2012). This advance in technology can be readily applied to other high complexity samples, such as metagenome shot-gun phage display libraries.

### Selective Metasecretome Phage Display

At present, published data on applications of phage display to aid metagenomic gene discovery is limited to “proof-of-principle” studies. T7 phage display and affinity capture was employed for enrichment and identification of genes encoding acyl carrier proteins and peptidyl carrier proteins from soil metagenome (Zhang et al., 2009). These proteins are essential components of biosynthetic enzymes (polyketide synthases and non-ribosomal peptide synthetases) that synthesize natural products with proposed antibiotic, immunosuppressant, and anticancer activities. After six iterative rounds of selection for enrichment of carrier proteins, a limited number (60) of phage clones were sequenced to evaluate usefulness of this approach for identification of carrier protein genes from large metagenomic DNA libraries. Display of the same soil metagenomic DNA library on the surface of filamentous phage and subsequent five rounds of panning did not yield any enrichment. Authors hypothesized that T7 phage display system is more suitable for functional selection of metagenome-encoded cytoplasmic enzymes because the T7 phage capsid protein is displayed on virions that are assembled in the cytoplasm and released by host cell lysis. In contrast, expression and display on filamentous phage depends on host secretion systems for translocation to the periplasm that is an oxidizing environment and therefore not suitable for most cytosolic enzymes which commonly contain an active site Cys residues.

**FIGURE 5:**
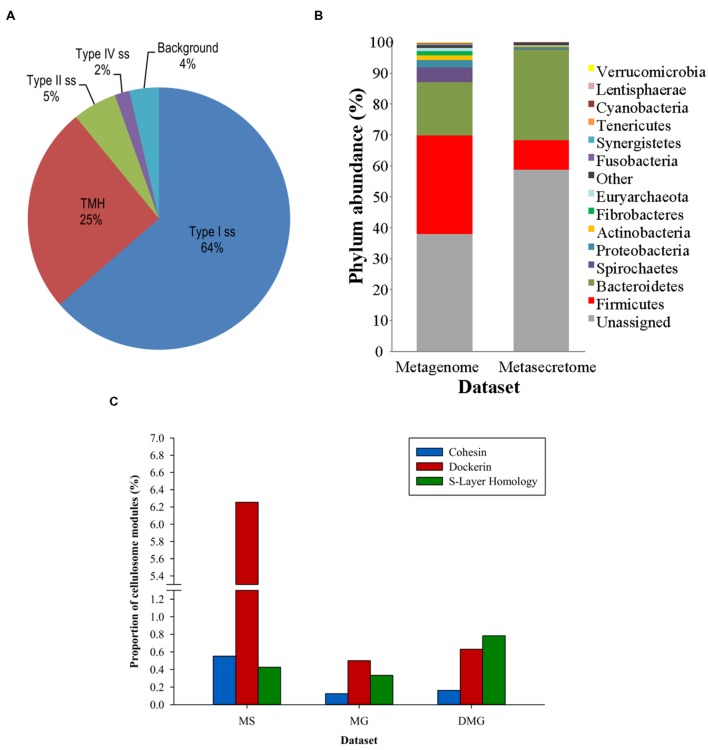
**Taxonomic distribution, signal sequence types, and cellulosome components enrichment in metasecretome phage display of fiber-adherent rumen microbial community. (A)** Distribution of signal sequence types and transmembrane helices in selected metasecretome recombinants; Abbreviations: Type I ss, classical signal sequence; Type II ss, lipoprotein signal sequence; Type IV ss, prepilin-like signal sequence; TMH, N-terminal or internal transmembrane α- helix/helices; background, ORFs encoding putative proteins without a predicted membrane-targeting signal/non-classical secretion, or ORFs encoding putative proteins and peptides ≤24 amino acid residues. **(B)** Taxonomic distribution of the phage-display-selected metasecretome; The taxonomic assignments, at the phylum level, were based on the distribution of the best BLASTP hits at a 30% amino acid sequence identity threshold for protein-coding genes predicted in metagenome and metasecretome datasets. Each section of the stacked columns represents the percentage of total protein-coding genes assigned to the corresponding phylum. The section labeled ‘Other’ contains putative protein-coding genes assigned to a phylogenetic group with low abundance in the dataset (<0.1%), while the section labeled ‘Unassigned’ corresponds to putative protein-coding genes with best BLASTP hit below 30% identity cut-off. **(C)** Enrichment for the components of cellulosome; Frequency of three putative distinct ‘signature’ cellulosome modules: cohesins (blue); dockerins (red) and surface S-layer homology (SLH) domains (green) in three datasets: MS, metasecretome dataset; MG, metagenome dataset and published deep-sequenced metagenome (DMG). The latter dataset is from (Hess et al., 2011). **(A,C)** are taken from (Ciric et al., 2014) and **(B)** from (Ciric, 2014) with permission.

Ciric et al. (2014) combined secretome-selective filamentous phage display (Jankovic et al., 2007) and NGS for the enrichment and sequence-based mining of bovine rumen plant-adherent microbial metagenome for fibrolityc enzymes (Ciric et al., 2014) (**Figure 4**). This approach enabled enrichment of metagenomic genes encoding surface, transmembrane and secreted proteins (metasecretome). Given that the selection relies on *E. coli* SecYEG translocons, this publication gave the first assessment of the selection system’s promiscuity, in particular for Gram-positive vs. Gram-negative secretion signals. Whereas secretome proteins from both types of bacteria have been selected for, taxonomic assignment of the sequenced inserts showed an increased frequency of secretome proteins from Gram-negative bacteria in the phylum Bacteroidetes (**Figure 5B**) relative to those from random shotgun sequence dataset. In terms of the type of secretion signals selected for at the metasecretome scale, inserts containing recognition sites for type I signal sequences were the most prominent, similar to what was reported for the genome-scale secretome selection (**Figure 5A**).

Next-generation-sequencing analysis of the metasecretome identified an increased frequency of putative ORFs encoding catalytic and binding modules of fibrolytic enzymes, as well as large numbers of putative modules (cohesins and dockerins) that are constructing blocks of cell-surface organelles called cellulosomes (Bayer et al., 2004), specialized for recognition and degradation of plant fiber, were detected (**Figure 5C**). A high proportion and taxonomic variety of cellulosomal modules, particularly those from cohesins and dockerins has not been reported in previously published metagenomic studies of the rumen microbiome, suggesting that phage display could be a powerful method for enrichment, display and identification of these modules which subsequently can be used in building of microbial “designer” fiber-degrading hyper structures. Genes encoding putative fibrolytic enzyme modules and cellulosome modules are candidates for functional characterization *via* affinity screening of methagenomic shot-gun phage display libraries.

Despite its power, there are several shortcomings of the metasecretome phage display approach, some of which could potentially be overcome with new and upcoming technologies. As is the case with the genome-scale secretome phage display, the metasecretome library is biased toward the sequences encoding the N-terminal portion of the secretome proteins, hence representation of the C-terminal domains is low. This can in principle be overcome by constructing fosmid- or cosmid-based phage display vectors that would have a capacity for large inserts (up to 40 kb) and would therefore be able to accommodate large portions of secretome proteins fused to pIII.

Next-generation sequencing using 454 or Illumina technologies is powerful in covering the totality of the meta secretome library inserts. However, due to the short lengths of templates required for these sequencing technologies and a PCR amplification step that is necessary in order to produce the sequencing library, sequence information is disconnected from the physical recombinant clones from which it was derived (Ciric et al., 2014), making it impossible to form a clone library without the standard Sanger sequencing. This issue, however, can be overcome by using novel sequencing technologies, like PACBio, which use long templates (Fichot and Norman, 2013; Wang et al., 2015) and could, in principle, be used in an approach that would allow tracking of a sequence to the template. This approach for sequencing could be combined with subtractive or normalization methods to enrich the library for the rare recombinant sequences (Gagic et al., 2015), and to build a balanced metasecretome clone bank that can be used as a resource for expression and purification of large number of secretome proteins from a microbial community, by virtue of their display on PPs.

A shot-gun metagenomic phage display libraries in Ff and T7 have potential to provide the widest possible coverage of SecYEG-dependent and –independent secretome proteins for affinity selection against complex or simple targets of interest to identify adhesins, secreted virulence factors or enzymes, whereas the metasecretome libraries provide a good source of data for large scale identification of immunogenic peptides. Amenability of phage display to large-scale, high-throughput screening using NGS and emerging phage display-based methods for enrichment of target genes in metagenomes, indicate that this is just a beginning of wider use of phage display to accelerate target gene discovery in metagenomics.

## Concluding Remarks

Phage display on Ff, combined with recent developments in sequencing technologies, provides a powerful approach for discovery of novel secretome proteins in variety of microorganisms and in microbial communities.

## Author Contributions

DG (50%), MC (30%), WW (5%) FN (5%), and JR (10%) have written the manuscript.

## Conflict of Interest Statement

The authors declare that the research was conducted in the absence of any commercial or financial relationships that could be construed as a potential conflict of interest.
