# Corrigendum: Exploring the Secretomes of Microbes and Microbial Communities Using Filamentous Phage Display

**DOI:** 10.3389/fmicb.2016.00927

**Published:** 2016-06-14

**Authors:** Dragana Gagic, Milica Ciric, Wesley X. Wen, Filomena Ng, Jasna Rakonjac

**Affiliations:** ^1^Institute of Fundamental Sciences, Massey UniversityPalmerston North, New Zealand; ^2^Animal Science, Grasslands Research Centre, AgResearch Ltd.Palmerston North, New Zealand

**Keywords:** phage display, secretome, adhesins, metagenomics, next generation sequencing, bacteriophage

Reason for Corrigendum:

In the original article, we have noticed that the affiliation is listed wrongly for Filomena Ng as only 2 (Animal Science, Grasslands Research Centre, AgResearch Ltd., Palmerston North, New Zealand). The correct affiliation should be Filomena Ng^1, 2^ (1-Institute of Fundamental Sciences, Massey University, Palmerston North, New Zealand; 2-Animal Science, Grasslands Research Centre, AgResearch Ltd, Palmerston North, New Zealand). The authors apologize for this oversight. This error does not change the scientific conclusions of the article in any way.

## Author contributions

MC, WW, FN, JR, and DG approved the content of Corrigendum.

### Conflict of interest statement

The authors declare that the research was conducted in the absence of any commercial or financial relationships that could be construed as a potential conflict of interest.

